# Subglottic Thyroglossal Cyst: A Case Report 

**DOI:** 10.22038/IJORL.2022.59977.3067

**Published:** 2022-09

**Authors:** Gayathri Vijayakumar, Nazrin Hameed, Preethy Mary, Mathew Dominic

**Affiliations:** 1 *Department of Ear Nose and Throat,* *Medical Trust Hospital, Kochi- 682016, Kerala, India.*

**Keywords:** Embryology, Intralaryngeal, Thyroglossal duct cyst

## Abstract

**Introduction:**

Thyroglossal duct cysts are a common congenital anomaly in the neck which usually present in adulthood as a midline neck swelling the location of which can vary from lingual, suprahyoid, infrahyoid and suprasternal.

**Case Report:**

Here we have described the case of a thirty-year-old male who presented with a history of recent onset dysphonia and an unnoticed neck swelling. Clinical examination revealed a midline 2 x 2 cm cystic lesion in the neck. On further evaluation using laryngoscopy and computed tomography the patient was found to have a smooth mucosa-covered cyst in the subglottic location. Surgical excision of the cyst was done via laryngofissure and the postoperative course was uneventful. The postoperative biopsy revealed a subglottic thyroglossal cyst.

**Conclusions:**

Though there have been reports of intralaryngeal extension of thyroglossal duct cysts, the subglottic location is extremely rare. Through this case report, we would like to highlight the atypical presentation of thyroglossal duct cysts and how an innocuous pathology can turn potentially life-threatening. We would also recommend avoiding a fine needle aspiration cytology in such cases due to the critical location.

## Introduction

Thyroglossal duct cyst is a developmental anomaly found in 7% of the population (1). Although thyroglossal duct cysts are the most common congenital abnormality in the neck, 50% are not diagnosed until adulthood (2). It most commonly presents as a cystic midline neck swelling which moves with deglutition and with protrusion of the tongue. While there have been reports of intralaryngeal thyroglossal cysts, location in the subglottic subsite is a rarity and to the best of our knowledge, there has only been one case of subglottic thyroglossal cyst in an adult which has been reported previously in English literature (3).

## Case Report

**Fig 1:**
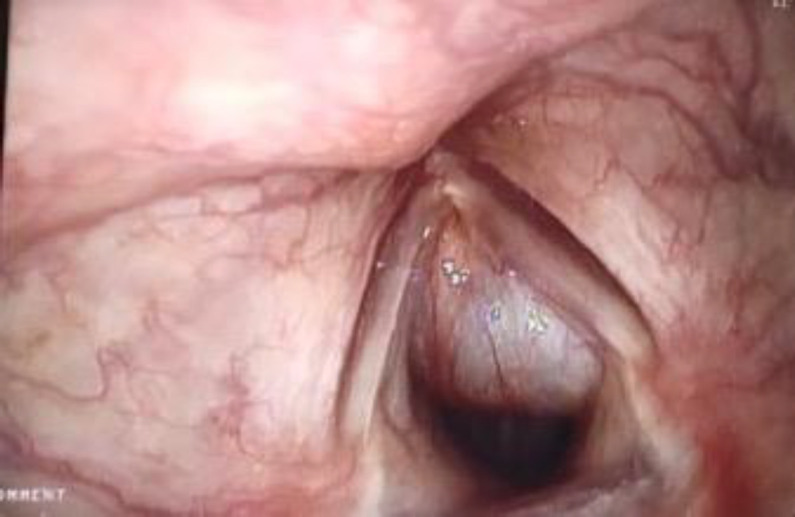
laryngoscopic picture showing an anterior subglottic cyst.

A thirty-year-old male presented to the outpatient department with a one-month history of dysphonia, following an episode of upper respiratory tract infection. He was a nonsmoker and had no history of throat pain, cough, difficulty in breathing or swallowing. On clinical examination, there was a 2x2 cm midline neck swelling which was unnoticed by the patient. The swelling was a well-defined cystic swelling which moved with deglutition and with protrusion of the tongue. Laryngoscopy revealed an anterior subglottic submucosal cyst with mobile vocal cords (Fig 1)

**Fig 2:**
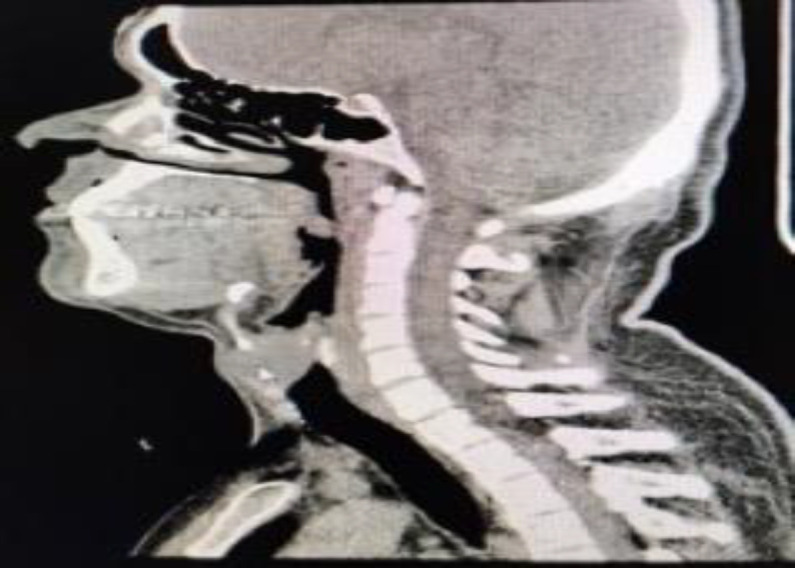
Computed tomography showing the cyst between the thyroid and cricoid cartilage with projection into the airway lumen

Computed tomography revealed an ovoid well-defined non-enhancing cystic lesion in the anterior midline of the neck of size 2.5x2.8x2.1cm between the thyroid and cricoid cartilage with scalloping of the superior aspect of the cricoid. Posteriorly the lesion projected into the subglottic region and upper trachea causing mild compromise of the airway. Due to the critical location of the cyst a Fine needle aspiration cytology (FNAC) was not performed in this case (Fig 2).

**Fig 3:**
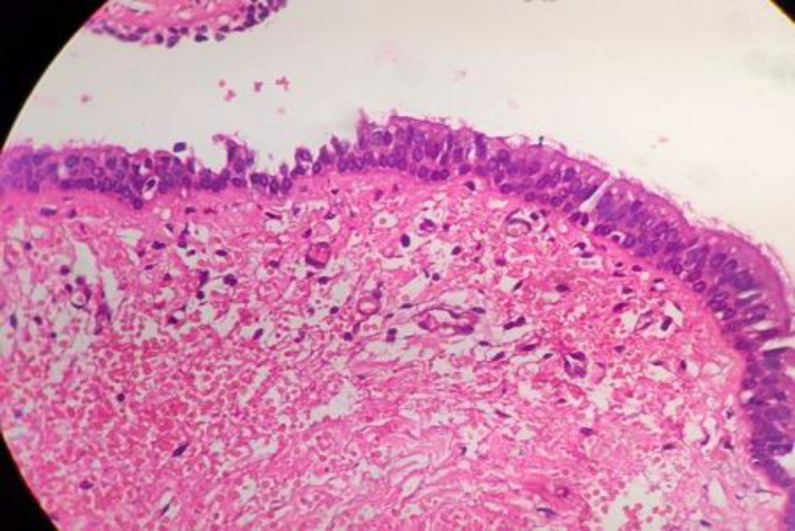
Histopathology suggestive of thyroglossal duct cyst

He underwent laryngofissure with cyst excision under general anaesthesia. A 2x2 centimetre cystic swelling was delineated by methylene blue dye injection in the midline between the thyroid and cricoid cartilage extending posteriorly to the ala of the thyroid cartilage. The entire cyst was excised in toto without any complications and was sent for histopathologic examination. Gross examination revealed a dye-stained collapsed and cut opened cystic specimen measuring 2x1.5x1cm. Microscopy revealed a cyst wall lined by respiratory epithelial lining with focal squamous metaplasia. Foci of ulceration showing granulation tissue were noted. The wall was fibro collagenous with few blood vessels, mild mixed inflammatory cell infiltrate and occasional glands. There were focal fibrosis and myxoid changes. The histopathologic examination was consistent with a thyroglossal cyst (Fig 3). 

The patient did not have any postoperative complications and was discharged on oral antibiotics on postoperative day 2. Following surgery patient is asymptomatic and on regular follow-up.

## Discussion

During the fourth week of fetal development, the thyroglossal duct develops from the epithelium on the floor of the pharynx at the site of the foramen caecum which lies at the junction of the anterior two-thirds and posterior one-third of the tongue. This duct descends caudally through the neck, in the midline anterior to the hyoid bone and the bilobed distal end differentiates into the thyroid. This duct normally undergoes involution or atrophy by the eighth or tenth week of intrauterine life. The incomplete involution of the thyroglossal duct remnant during embryogenesis leads to the formation of a thyroglossal duct cyst (TDC). It can be formed anywhere along its pathway of descent from the foramen caecum to the suprasternum and the most common presentation is that of an anterior neck swelling that moves with deglutition and with protrusion of the tongue. Although most are situated in the midline, 10 to 24% of the cysts are located laterally, usually to the left (4). 

This has been explained by the fact that levator glandulae thyroideae muscle is ordinarily found to the left. A lateral position may be expected especially in the region of the thyroid cartilage because the shape of the thyroid cartilage is like the prow of a ship, on which the cyst cannot remain. 

The tract may remain silent for several years and may present symptomatically due to inflammation, infection or mucous retention (5). According to a study by Allard the various locations of a thyroglossal cyst can be infrahyoid (60.9%), suprahyoid (24.1%), lingual (2.1%) and suprasternal (12.9%) (4). An infrahyoid thyroglossal cyst may also expand to involve the larynx, although this is a very rare presentation. In our case, it presented as a mucosa-covered subglottic cyst which caused dysphonia following an upper respiratory infection. Although a benign mass, malignant change in the cyst or an airway obstruction due to an intralaryngeal thyroglossal duct cyst can cause the disease to show a fatal cause (6). This ascertains the need for considering atypical presentations of thyroglossal duct cysts and the role of computed tomography and histopathology in confirming the same in cases where it may mimic a laryngeal cyst. Although FNAC is an accepted preoperative investigation for thyroglossal cyst, in our case we did not subject the patient to FNAC due to its critical location. Through this example, we would also like to stress on the importance of performing imaging prior to FNAC and how an FNAC may be detrimental in a subglottic thyroglossal cyst by potentially causing airway compromise.

## Conclusion

Through this case report we would like to highlight the atypical presentation of thyroglossal duct cysts and besides its malignant potential, how an innocuous pathology can turn potentially life-threatening by causing airway compromise if ignored. Thus thyroglossal duct cyst should be considered as a differential diagnosis of subglottic cysts and FNAC should be deferred in such cases due to the risk of airway obstruction. 
